# Emerging Technologies Tackling Adeno-Associated Viruses (AAV) Immunogenicity in Gene Therapy Applications

**DOI:** 10.3390/pharmaceutics17111492

**Published:** 2025-11-19

**Authors:** Tatiana Egorova, Anna Starikova, Anna Polikarpova

**Affiliations:** 1 Laboratory of Modeling and Therapy of Hereditary Diseases, Institute of Gene Biology Russian Academy of Sciences, 119334 Moscow, Russia; egorovatv@genebiology.ru (T.E.); a.alexandrova.marlin@gmail.com (A.S.); 2 Marlin Biotech LLC, 354340 Sochi, Russia

**Keywords:** adeno-associated virus, gene therapy, immune response, neutralizing antibodies

## Abstract

Adeno-associated viral vectors have proven to be a safe and effective gene therapy delivery system. Over the past decade, the approval of AAV gene therapies made a revolution in treatment of severe hereditary diseases, including spinal muscular atrophy, AADC deficiency, and others. Recombinant AAV-based therapeutics are currently intended for single administration. Safety concerns arise from immune responses to AAV and the resulting transgene, which can render subsequent injections ineffective. It remains unclear whether patients who have received an AAV-based gene therapy will need re-administration in the future. Furthermore, since many people have neutralizing antibodies or memory T cells against AAV from natural infections, it is crucial to overcome pre-existing immunity. This review considers existing modern approaches aimed to overcome both pre-existing natural immunity and immunity obtained after the administration of a gene therapy drug, which include various modifications of the viral drug (capsid modification, codon optimization), the use of empty capsid traps, and pharmacological support (immunosuppressive corticosteroids, inhibitors of various branches of the immune response, nanoparticles, IgG-degrading enzymes). The goal of this review is to illustrate the importance of this challenge and to highlight potential strategies for overcoming immunity to AAV-based gene therapies, contributing to the development of a successful therapeutic gene delivery platform.

## 1. Introduction

Among the wide variety of viruses that can infect mammalian cells and can be used as vectors for gene delivery, adeno-associated viruses (AAVs) are of particular interest for gene therapy because they do not cause diseases in humans and animals, have tropism for most cells and tissues, and are characterized by high transduction efficiency [1]. AAV-based drugs that can deliver a transgene to various target tissues have been actively developed over the past two decades. Currently, AAV-based drugs for the treatment of spinal muscular atrophy (SMA), Duchenne muscular dystrophy (DMD), hemophilia B, and a number of other diseases have received regulatory approval [2]. A significantly larger number of other AAV gene therapies are in various stages of clinical trials for the treatment of genetic and acquired diseases. The clinical implementation of AAV-based gene therapy has prolonged life and enhanced the quality of life for numerous patients suffering from fatal diseases. The ability of AAV vectors like AAV9 to cross the blood–brain barrier is enabling applications in CNS disorders, such as SMA. Clinical trials are also exploring AAV-based treatments for various pediatric conditions, including ornithine transcarbamylase deficiency, glucose transporter I deficiency, and Niemann–Pick disease type C. AAV-based therapies are moving beyond ophthalmological, neuromuscular, and hematological conditions into a wider array of monogenic disorders, including those affecting the nervous system and metabolic pathways. The efficiency of gene transfer and the possibility of providing long-term therapy make AAV vector systems very attractive. In fact, AAV-based gene therapy has the potential to treat and even resolve diseases for which there was previously no cure or only supportive treatment. However, despite the advantages of AAVs, their use as vectors for gene delivery in clinical settings has serious limitations related to immune system responses.

Innate and adaptive immune responses to these vectors and their transgene products represent a significant obstacle to their wider use in patients. It has been shown that activation of cellular and humoral immunity is often induced upon exposure to wild-type AAV, resulting in the formation of specific neutralizing antibodies [3]. Pre-existing neutralizing antibodies to the AAV vector may partially or entirely inhibit the transduction of target cells, negatively impacting the efficacy of gene therapy. Consequently, patients with AAV antibodies in their bloodstream are presently excluded from clinical trials concerning systemic delivery of AAV-based therapies, thereby diminishing the pool of patients eligible for this life-saving treatment [4]. Antibody-mediated complement activation can cause inflammatory reactions, decreased transduction efficiency, and even serious adverse reactions. The most common adverse events reported in AAV clinical trials were thrombotic microangiopathy and acute systemic inflammation.

Administration of the viral vector results in the formation of a robust humoral response in both patients and animal models, which in turn prevents repeated administration of AAV-based drugs. In contrast to the humoral immune response, studying cellular immunity to both AAV capsid or therapeutic proteins is more challenging due to the difficulty of reproducing these responses in animal models. Several cases of immune response to the transgene have been reported in clinical trials [5]. Due to the lack of tolerance to the target protein, gene therapy for diseases caused by recessive protein-deficient mutations may be more likely to induce immunity to the transgene. Numerous studies in mice, dogs, and non-human primates have demonstrated stable transgene expression in target tissues after drug administration without activation of the T-cell immune response [6]. In contrast to animal models, administration of viral vectors to patients with acquired immunity resulted in reactivation of memory T cells and destruction of virus-infected cells by cytotoxic CD8+ T lymphocytes, which shortened the duration of transgene expression. T-cell activation and associated hepatotoxicity have been observed in patients in clinical trials involving systemic administration of AAV-based drugs [7]. Some pre-clinical studies show that even single amino acid variations can be recognized by the host immune system [5].

In the case of neuromuscular diseases, efficient delivery of the therapeutic construct requires systemic administration of a huge dose of recombinant viruses due to the large muscle mass. In addition to systemic side effects, destruction of transduced muscle fibers by cytotoxic T lymphocytes may result from an immune response to the therapeutic protein, rendering gene therapy ineffective. Where possible, alternative routes of administration may be considered instead of systemic injections to reduce subsequent immune reactions. For example, Leber’s amaurosis is caused by a mutation in the RPE65 gene, which is expressed exclusively in the retinal pigment epithelium. Gene replacement therapy for Leber’s amaurosis requires injection of RPE65 into the eye. The recently approved drug voretigene neparvovec-rzil for subretinal injection has demonstrated efficacy in clinical trials. The weak immune response observed after its administration can be explained by the local injection in an isolated immune-privileged environment [8].

Despite the impressive success in treating genetic diseases with approved gene therapies and the promising results of ongoing clinical trials, serious adverse reactions and deaths have been observed following the administration of gene therapy drugs [9]. Studies reveal that both innate and adaptive responses triggered by the AAV capsid, along with humoral and cellular responses elicited by transgene products, significantly contribute to the risks associated with the systemic administration of AAV-based gene therapy. While previous comprehensive reviews have extensively detailed the immunogenic mechanisms of AAV vectors, this review uniquely concentrates on recent advances in clinical strategies and practical approaches for overcoming pre-existing and induced immunity in therapeutic settings. In this review, we provide an overview of immune responses directed against the AAV capsid and transgene, and we summarize current scientific and clinical data on strategies to overcome pre-existing immunity to AAV. This immunity can be acquired both through natural infection and following the first administration of an AAV-based therapy. The review is divided into sections that briefly describe how the immune response to AAV develops and explore the mechanisms that may help in overcoming different branches of immunity. We discuss cases and diseases where AAV administration is required for seropositive patients and outline methods for overcoming immunity to the transgene and capsid. Our review not only addresses potential approaches for evading the humoral and cellular immune responses to AAV but also highlights methods already employed in clinical practice.

## 2. Immunity to AAV-Based Drugs

**Figure 1:**
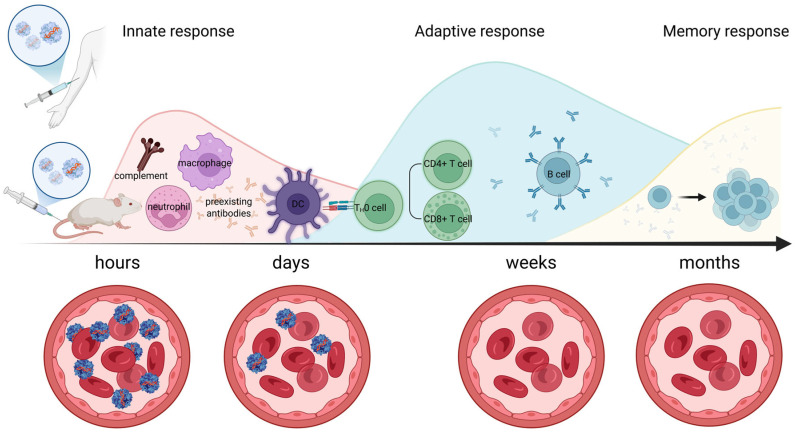
Timeline of immune activation following AAV administration. Innate Response (Hours): The initial hours post-administration involve innate immunity activation. This includes recognition by pattern recognition receptors, activation of the complement system, and the recruitment of inflammatory cells. Key components like macrophages and dendritic cells are depicted engaging with the AAV particles. Adaptive Response (Days to Weeks): Following the innate response, adaptive immunity is engaged. Over days to weeks, T cells (both helper CD^4+^ and cytotoxic CD^8+^ T cells) become activated and specific antibodies against AAV capsids are produced by B cells, leading to neutralization of the vectors. Memory Response (Months): Long-term, memory immune cells are formed, leading to a rapid and robust response upon re-exposure. This phase is crucial for understanding pre-existing immunity in treated patients. Created in Biorender. Egorova, T. (2025) https://BioRender.com/mwqw4pe (accessed on 17 July 2025).

Immunity to adeno-associated virus involves both innate and adaptive immune responses, with complement activation playing an important role, particularly in high-dose systemic gene therapy (Figure 1). The innate immune mechanisms mediate the initial non-specific response, while the adaptive system generates specific long-term memory through interactions between B and T cells. Complement, which is part of the innate system, can be activated by the AAV capsid, resulting in inflammation and the formation of neutralizing antibodies, which are key components of the adaptive response.

### 2.1. AAV and Innate Immunity

When pathogens, including viral vectors used for gene therapy, enter the body, non-specific activation of the innate immune system occurs, which subsequently stimulates the development of adaptive immune responses to AAV. All pathogens carry pathogen-associated molecular patterns (PAMPs) that are recognized by pattern-recognizing receptors (PRRs) present on the immune cells such as macrophages/monocytes, B lymphocytes, and antigen-presenting cells. PRRs are classified by ligand specificity, function, and localization into several groups, namely Toll-like receptors (TLRs), Nod-like receptors, complement receptors, RIG-I-like receptor helicases, and C-type lectin receptors [10]. Recognition of a PAMP by the corresponding receptor triggers intracellular signaling pathways that lead to activation of the transcription factor NF-κB, expression of major histocompatibility complex (MHC) genes, and secretion of proinflammatory cytokines and type I and III interferons (IFNs) [11]. Secreted interferons and cytokines mediate the expression of genes that limit viral replication and also stimulate immune memory cells [12].

TLR-mediated activation of innate immunity is an important mechanism of AAV recognition. TLRs are present on the membranes of leukocytes including dendritic cells, macrophages, natural killer cells, cells of adaptive immunity (T cells and B cells), and non-immune cells (epithelial cells, endothelial cells, and fibroblasts). Studies have shown that intracellular TLR9 promotes immune responses against the transgene and capsid [13]. TLR9 presented on macrophages and dendritic cells specifically recognizes unmethylated cytosine–guanine dinucleotides (CpG) within the vector DNA. Preventing TLR9 signaling by reducing the number of CpG dinucleotides in the AAV genome has been shown to enhance AAV-mediated gene expression. CpG motifs are found in both the expression cassette, including promoter and CDS, and the ITRs. Minimizing CpG motifs in both the vector genome and promoter regions could enhance the safety and efficacy of gene therapy. The recent study by Li et al. [14] examined strategies to evade innate immune responses using AAV1 vectors. They compared a CpG-free OVA expression cassette, featuring a CMV enhancer and EF1α promoter, with a CK8-driven cassette. Their findings demonstrated that depleting CpG motifs in vector genomes and incorporating TLR9 inhibitory sequences work synergistically to reduce immune activation. When combined with a muscle-specific promoter, this approach resulted in sustained transgene expression in muscle tissue while eliciting minimal local or systemic CD^8+^ T-cell responses. Similarly, CpG deletion resulted in a significant reduction in CD^8+^ T-cell infiltration when injected intramuscularly into hemophilia B mice [15]. Reducing the number of CpG motifs in promoter sequences is an attractive strategy. Among the synthetic promoters used for muscle-specific expression, the Spc5-12 promoter is entirely CpG-free [16]. Ongoing clinical trials (NCT05693142, Regenxbio, Rockville, MD, USA; GNT-016-MDYF, Genethon, Évry-Courcouronnes, France) are evaluating this promoter for DMD treatment, with results still pending. Removing CpGs from the ITRs may further decrease AAV immunogenicity. A recent mouse study demonstrated that CpG depletion from the ITRs is possible without compromising the vector’s biological activity [17]. However, this modification resulted in lower vector titers, and it remains unclear whether it reduces inflammatory responses against rAAV in vivo. Overall, these insights suggest promising avenues for improving gene therapy efficacy by modulating innate immune activation.

There is an interesting hypothesis that, in addition to viral DNA, innate immunity to AAV is also activated by double-stranded RNA. dsRNAs produced by ITR promoter activity accumulate and stimulate the MDA5 sensor in AAV-infected hepatocytes, leading to the expression of type I interferon. It has been shown that inhibition of MDA5 reduces the amount of interferon and increases the level of transgene expression in vitro [18]. Clinical trials have not yet confirmed this hypothesis, but it may explain why in some cases activation of the cellular immune response is observed several weeks after AAV administration, which corresponds to dsRNA synthesis [19]. Recent studies highlight that m6A modifications within the body of transcripts have been shown to protect dsRNA from recognition and subsequent activation of MDA5. These mechanisms are supported by research using synthetic poly (I:C), a compound that mimics viral dsRNA and is used to study innate immune responses; modifications such as methylation or structural changes in poly (I:C) can suppress MDA5 activation and downstream immune signaling [20]. Incorporating these findings emphasizes that the activation of MDA5 by dsRNA can be mitigated through various structural modifications, which is an important consideration in understanding innate immune evasion during viral infections and gene therapy.

Innate immune reactions to AAV can lead to tissue inflammation and damage, which adversely affects the functioning of organs such as the heart, liver, and central nervous system. The activation of innate immunity to the AAV capsid can also provoke the adaptive immune response, creating a constant cycle of immune activation and organ and tissue damage.

In pre-clinical development, a number of strategies to circumvent the innate immune response were investigated and implemented in clinical settings. These approaches offer the potential to reach more patients for whom gene therapy could be the preferred, if not the only, treatment option. They can be divided into two main classes: (1) AAV drug modification and (2) host immune suppression [21]. Modifying the capsids is an effective strategy for overcoming innate immunity including encapsidation into exosomes, which facilitates immune evasion. The viral genome can also be optimized to minimize immune induction, which occurs through CpG reduction. Pharmacological immunosuppression is currently widely used in clinical practice. Corticosteroids (prednisolone, deflazacort) bind to glucocorticoid receptors and alter transcriptional signaling, leading to a decreased level of TLR expression, suppression of proinflammatory cytokines, and upregulation of anti-inflammatory cytokines. This leads to a global anti-inflammatory and immunosuppressive effect [22].

### 2.2. AAV and Complement Activation

The complement system is a complex of protective proteins that are constantly present in blood. It is a cascade system of proteolytic enzymes designed for humoral protection of the body from pathogens. The complement system is an important component of innate immunity. There are three main pathways for activating the complement system: classical, alternative, and lectin. The classical complement pathway requires opsonization of the foreign cell by antibodies, while the alternative and lectin pathways can be activated in the absence of antibodies [23]. There is evidence that both the classical and alternative complement pathways can be initiated following AAV infection [24]. Early in vitro studies showed that AAV-induced complement activation was observed exclusively in seropositive human serum. An antibody-dependent mechanism of in vitro complement activation by AAV was supported by the demonstration that IgG depletion or serum pre-treatment with an IgG-degrading enzyme prevented AAV-induced complement activation [25]. Overall, these results demonstrate the important role of pre-existing neutralizing antibodies in complement activation.

Different AAV serotypes have been shown to have different susceptibility to complement activation. At least for antibody-mediated complement activation, this may depend on the presence of pre-existing antibodies and affinity for rAAV. Moreover, the direct interaction between AAV particles and complement proteins has been demonstrated, suggesting that protein sequences and spatial structure may influence the strength of this interaction [26,27]. A recent study provides experimental evidence for differences in complement activation for AAV serotypes 2 and 8 [28].

The complement system is both highly reactive and tightly regulated, enabling it to respond swiftly to pathogens while preventing overactivation that could cause unintended damage to host tissues. Despite this, in clinical trials involving high-dose (equal to or exceeding 1 × 10^13^ vg/kg) AAV-based therapies, excessive or uncontrolled activation of the complement system often develops, which leads to a variety of human pathologies, including the rare complement-mediated form of thrombotic microangiopathy and atypical hemolytic uremic syndrome (aHUS) [26]. In the recent DMD trials and other related studies, increases in complement components like C3, C4, and C5b-9, along with decreases in platelet counts, serve as valuable indicators of complement activation and microangiopathy. In the Pfizer clinical trial (PF-06939926, NCT03362502), two boys experienced serious adverse events (SAEs) characterized by atypical hemolytic uremic syndrome (aHUS)-like complement activation, including a case of acute kidney injury. A hallmark of this immune response was the pattern of decreased platelets and reduced C4 levels observed within 14 days post-infusion (Salabarria et al., 2021 [29], presentation at MDA). Similarly, Solid Biosciences’ trial (SGT-001, NCT03368742) reported SAEs associated with complement activation [29]. In this study, three participants exhibited clinical signs of thrombotic microangiopathy, with rapid decreases in platelet counts observed within 7–10 days post-infusion. These changes were accompanied by depletion of C3 and C4 levels and an increase in C5b-9 around the 7th day, indicating robust complement system activation. These risks can be mitigated by using appropriate immunosuppressive strategies when administering AAV-based drugs to the patients. Adequate therapy with antibodies against complement proteins (e.g., eculizumab) has resulted in cure of patients [12].

### 2.3. AAV and Adaptive Immunity

Activation of adaptive immunity takes longer than the launch of innate programs. The advantages of the adaptive immune response are its antigen specificity and the ability to form immunological memory. There are two main mechanisms of adaptive immunity: humoral immunity (B cells and antibodies) and cellular immunity (cytotoxic CD^8+^ T lymphocytes). CD^4+^ T lymphocytes are key mediators of both humoral and cellular immunity, activating B cells and cytotoxic T lymphocytes.

After administration of AAV-based drugs, antigen-presenting cells (dendritic cells, macrophages, and others) capture viral particles by transduction or phagocytosis and present processed antigens on the major histocompatibility complex (MHC) to activate B or T lymphocytes. B lymphocytes recognize the antigen on MHC II and produce antibodies. Antibodies produced by B cells can act in different ways. Neutralizing antibodies (nAbs) prevent subsequent AAV entry into cells, such as upon repeated administration or upon initial administration after natural infection. Antibodies can coat the surface of the virus, marking it for phagocytosis, or bind to viral particles, leading to activation of the classical complement pathway. The humoral immune response, in particular nAb production, is one of the most effective barriers to successful gene delivery using AAV vectors. Following AAV transduction, antigens are presented to CD^4+^ T-helper cells, leading to B-cell maturation, expansion, class switching, and increased production of antibodies against the AAV capsid [12].

In addition to the humoral immune response, the T-cell response plays an important role in the effectiveness of AAV gene therapy. Activation of cytotoxic CD^8+^ T lymphocytes with the antigen presented on MHC I is carried out by CD^4+^ T helpers and leads to the destruction of AAV-infected cells by secreting granzyme, perforin, and inflammatory cytokines. However, CD^4+^ T helpers themselves can also participate in the development of the immune response by producing cytokines and inducing inflammation [30]. The T-cell response was first detected in patients with hemophilia in clinical trials of an AAV-based drug carrying clotting factor IX. The immune response to the drug resulted in elevated liver enzymes and loss of transgene expression in liver cells [31]. In contrast, administration of lower doses of AAV resulted in the appearance of capsid-specific T cells, but no increase in liver transaminases or loss of transgene expression was observed [32]. Further studies confirmed that the capsid-specific immune response is dose-dependent, with low doses of AAV more likely to cause low-grade inflammation, which is easily corrected by adequate immunosuppression [33]. Daniel J Hui and colleagues [34] identified and characterized CD^8+^ T-cell epitopes on the AAV capsid, including AAV1, 2, and 8, from spleens of pediatric and adult human subjects. Their findings revealed that several identified epitopes across different AAV serotypes showed a high degree of conservation. Such insights could aid in the development of less immunogenic AAV vectors, employing approaches similar to those used with MHC class II epitope modification in therapeutic proteins [35,36], which have shown success in reducing AAV immunogenicity in murine models. In addition to the T-cell response to the AAV vector capsid, activation of cellular immunity can also occur in response to the transgene. In clinical trials of AAV2-based minidystrophin no transgene expression was observed after intramuscular delivery of a functional transgene to patients with DMD. Dystrophin-specific T-cell immune responses were detected after treatment [37]. Activation of CD^8+^ T cells against muscle cells may trigger persistent inflammation and loss of transduced fibers, making gene therapy futile for patients. The possibility of destruction of transduced muscle cells by T lymphocytes directed against foreign epitopes within the therapeutic transgene has been shown [38]. Very similar suspicious unexpected serious adverse reactions were observed in five patients with DMD, enrolled in three AAV-based gene therapy clinical trials (NCT04281485 [39], NCT04626674 [40], and 2020-002093-27 [41]). Symptoms suggestive of an immune response appeared 3–6 weeks after administration: all patients had severe limb muscle weakness and loss of ambulation, as well as respiratory muscle weakness requiring temporary respiratory support. The timing of these adverse reactions was consistent with expression of the therapeutic transgene, and laboratory data indicated a cytotoxic T-cell immune response against the delivered microdystrophin. Adverse reactions were treated with immunomodulatory agents and plasmapheresis. To effectively mitigate cytotoxic CD^8+^ T-lymphocyte activation in gene therapy, the following therapeutic strategies are employed. Calcineurin inhibitors like tacrolimus and cyclosporine A suppress T-cell activation by inhibiting proinflammatory cytokines such as IL-2, reducing CD^8+^ T-cell proliferation and cytotoxicity. Sirolimus inhibits the mTOR pathway, not only dampening T- and B-cell activation but also promoting regulatory T-cell (Treg) induction, which helps establish immune tolerance (e.g., NCT02240407). However, it is important to note that cyclosporine A may exhibit immunostimulatory effects in certain contexts, highlighting the need for combination strategies [42].

Generating an immune response against a foreign protein delivered by AAV is a critical issue in gene replacement therapy for DMD. Microdystrophin epitopes are recognized as foreign in DMD patients, resulting in immune activation. One therapeutic approach for these patients is utrophin-based therapies. An advantage of utrophin is its non-immunogenicity, achieved through expression in the thymus during the early stages of embryogenesis [43]. Studies demonstrate lower immunogenicity of microutrophins compared to microdystrophins [44]. Delivery of murine microutrophin results in a lower rate of CD^8+^ CTL infiltration into the muscle of mouse models compared to other transgene products [45]. Thus, the use of AAV to deliver self-produced proteins as transgenes shows promise as a gene therapy approach.

Monitoring biomarkers like cytokine panels, complement activation products (e.g., C3a, C5a), and T-cell assays in AAV gene therapy is crucial for predicting and managing adverse immune events, such as liver toxicity and inflammatory responses. These biomarkers help identify patients at risk, guide the use of immunosuppressive drugs like corticosteroids, and improve overall patient safety by providing real-time insights into the body’s immune response to the AAV vector. 

## 3. Overcome Pre-Existing Immunity to AAV

All currently approved gene therapies use naturally occurring AAV serotypes for transgene delivery (Table 1) [46]. During life, each person may encounter a natural AAV infection and thus become pre-immunized and generate both humoral and T-cell immune responses to rAAV. Circulating neutralizing antibodies can directly affect the efficacy of AAV therapy and prevent transduction of target tissues. It is estimated that up to 80% of healthy adults are seropositive and have antibodies to at least one naturally occurring AAV serotype [47,48]. This number may vary depending on, for example, age or region of analysis. In addition to neutralizing antibodies, circulating antibodies that bind rAAV particles may also reduce tissue transduction due to opsonization of vectors that facilitate Fc-mediated clearance. The highest percentage of both binding and neutralizing antibodies in the human population is shown for AAV serotypes 1 and 2 and the lowest for AAV serotypes 5, 8, and 9. It is also known that some neutralizing antibodies can cross-react with other serotypes [47].

In current practice, approved gene therapies cannot be administered to seropositive patients and they are also excluded from clinical trials. Due to the ease of laboratory testing, pre-existing immunity to AAV is assessed according to the circulating antibody titer. An acceptable cut-off value is defined for each specific clinical trial and there is variability in antibody assessment methods used [49]. The total binding antibody titer is defined by ELISA-based screenings or dot-blot assay in laboratory settings. Currently, quantitative antibody binding remains the most sensitive and standardized method. For various versions of this assay, capsid particles of the selected serotype are immobilized on a plate and coated with various dilutions of the patient’s serum. Antibodies present in the serum are then detected using enzyme-conjugated human-specific secondary antibodies for further visualization. Assay results are presented as a 1:X ratio, where X is the last serum dilution at which the reaction was confirmed. For approved gene therapies an acceptable titer is defined as less than 1:50 for onasemnogene abeparvovec-xioi (Zolgensma, Novartis Gene Therapies, Basel, Switzerland) and <1:400 for delandistrogene moxeparvovec-rokl (Elevydis, Sarepta Therautics, Cambridge, MA, USA). Fidanacogene elaparvovec-dzkt (BEQVEZ) and valoctocogene roxaparvovec-rvox (ROCTAVIAN) indications rely on results of FDA-approved companion diagnostic antibody quantification protocols [50]. Neutralizing antibody assessment relies on in vitro and in vivo transduction inhibition. Determining neutralizing antibody titers is highly sensitive to multiplicity of infection for a specific serotype and target cell culture. Additionally, transduction inhibitors other than Ig cannot be differentiated during such an assay. In etranacogene dezaparvovec-drlb (HEMGENIX) trials, an unvalidated clinical trial assay was utilized to assess pre-existing neutralizing anti-AAV5 antibodies [51]. The lack of standardized eligibility criteria for pre-existing AAV immunity across clinical trials introduces heterogeneity in safety and efficacy data, complicating cross-trial comparisons and potentially masking risks. For example, a study conducted in patients with hemophilia in the UK identified a subgroup of patients who had AAV-binding antibodies but were negative in transduction inhibition tests or vice versa [52]. Therefore, a patient excluded in one trial due to ELISA positivity might qualify for another. The rationale for a standardized method to measure pre-existing antibodies has been reviewed in depth by Mendell and colleagues [53].

Another safety consideration is connected with cellular immunity. Once formed, memory T cells can be activated during gene therapy. Memory T cells against AAV capsids can eliminate transduced cells, limiting long-term transgene expression. This can lead to loss of therapeutic effect or provoke immune-mediated toxicity and severe adverse reactions when attacking vital organs. Elevation of transaminases due to hepatocyte damage was demonstrated to be connected with T-cell proliferation [31]. Gene therapy protocols include monitoring of cellular immunity status and liver transaminase level after vector delivery. Cellular immunity is typically assessed using ELISpot, a fluorescence-based variant of ELISpot, or flow cytometry-based assays designed to detect cytokines in peripheral blood in response to specific antigens. The methodological aspects of these analytical platforms and experience with monitoring cellular responses in clinical trials are summarized in a recent review [54].

Little is known about memory cell prevalence in pre-treated patients and its correlation with transduction level, transgene expression, and gene therapy efficiency. Analysis of cellular immunity in healthy volunteers demonstrated the highest prevalence of memory T cells against the AAV9 serotype (49%) followed by AAV8, AAV4, AAV2, and AAVrh10 (24, 10, 9, and 7%, respectively) [55]. The authors did not find any correlation between detection of memory T cells and anti-AAV antibodies. Cross-reactivity of T cells was also recorded: 26% of the positive donors are reacting to two or more serotypes. Moreover, in-depth analysis of cytokine expression profile demonstrated that at least AAV9-recognizing T cells do not produce IL-2 that suggests they are unlikely to be cytotoxic effector T cells and instead could be a hallmark of a regulatory T-cell (Treg) population [55]. Functional significance of these findings should be further investigated.

**Table 1:** AAV-based gene therapy products approved for clinical use.

INN	Product Name	Disease	Manufacturer	Route of Administration	Dose	Serotype	Promoter	Approving Countries	Reference
Alipogene tiparvovec	Glybera	Familial lipoprotein lipase deficiency	UniQure	Intramuscular	1 × 10^12^ vg/kg	AAV2	CMV	European Union (2012)	[56]
Voretigene neparvovec-rzyl	Luxturna	Retinal dystrophy	Spark Therapeutics	Subretinal	1.5 × 10^11^ vg per eye	AAV2	CAG	United States (2017), European Union (2018), Australia (2020), Canada (2020), Switzerland (2020)	[57]
Onasemnogene abeparvovec-xioi	Zolgensma	Spinal muscular atrophy (SMA)	Novartis Gene Therapies	Intravenous	1.1 × 10^14^ vg/kg	AAV9	CAG	United States (2019), Israel (2019), European Union (2020), Japan (2020), Brazil (2020), Canada (2020), Australia (2021), United Kingdom (2021), Russia (2021), Singapore (2023)	[58,59,60,61]
Etranacogene dezaparvovec-drlb	Hemgenix	Hemophilia B	CSL Behring LLC	Intravenous	2 × 10^13^ vg/kg	AAV5	LP1	United States (2022), European Union (2023), United Kingdom (2023), Canada (2023), Switzerland (2023), Australia (2023)	[62]
Valoctocogene roxaparvovec-rvox	Roctavian	Hemophilia A	BioMarin Pharmaceutical Inc.	Intravenous	6 × 10^13^ vg/kg	AAV5	HLP	United States (2023)	[63,64]
Delandistrogene moxeparvovec-rokl	Elevidys	Duchenne muscular dystrophy (DMD)	Sarepta Therapeutics	Intravenous	1.33 × 10^14^ vg/kg	AAVrh74	MHCK7	United States (2023), Middle East (2025), Brazil (2024), Israel (2025), Japan (2025)	[65]
Eladocagene exuparvovec	Upstaza (EU) and Kebilidi (USA)	Aromatic L-amino acid decarboxylase deficiency	PTC Therapeutics	Intraputaminal	1.8 × 10^11^ vg total	AAV2	CMV	United States (2024), European Union and United Kingdom (2022), Brazil (2024)	[66]
Fidanacogene elaparvovec-dzkt	Beqvez	Hemophilia B	Pfizer Inc.	Intravenous	5 × 10^11^ vg/kg	AAVrh74	Liver-specific modified promoter	United States (2025), Canada (2025)	[67]

CMV—cytomegalovirus promoter; CAG—chicken beta-actin promoter; LP1—liver-specific promoter 1; HLP—hybrid liver-specific promoter; MHCK7—minimal muscle creatine kinase promoter.

**Figure 2:**
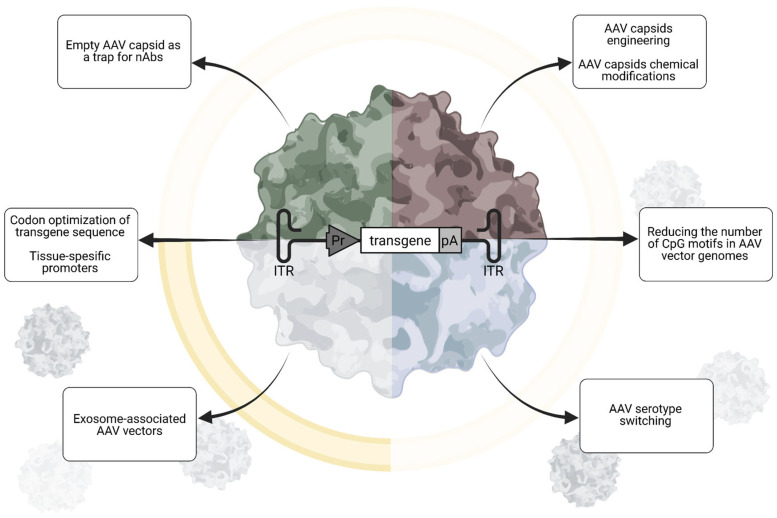
Strategies to overcome pre-existing immunity in AAV gene therapy aimed at modifying AAV: Utilizing empty capsid decoys, engineering or serotype switching of AAV capsids, reducing CpG motifs in AAV vector genomes, and packaging AAVs into exosomes. Created in Biorender. Egorova, T. (2025) https://BioRender.com/8chnaor (accessed on 17 July 2025).

Taking into account all the obstacles to determining pre-existing immunity and the suboptimal nature of the methods used, as well as all the shortcomings of the established eligibility criteria, it is now obvious that a significant group of patients cannot be treated with existing gene therapy drugs due to the high risk of complications. For example, 13% (23/173) of the examined patients were excluded from the randomized phase 3 EMBARK study of delanistrogene moxeparvovec [65]. Therefore, methods that allow curing seropositive patients with gene therapy products are in great demand. Several approaches are currently being developed to deliver genes in the presence of natural pre-existing immunity to the AAV capsid, including empty capsid decoys, capsid engineering/serotype switching, capsid chemical modification, or packaging capsids into exosomes (Figure 2) [68].

Delivery of capsids lacking the therapeutic transgene aims to reduce the neutralization capacity of pre-existing antibodies, thus inhibiting immune clearance of functional vectors and allowing them to reach target tissues [69]. In pilot work, Mingozzi and colleagues proposed to deliver empty capsid decoys of the same serotype along with therapeutic vectors. Empty capsids acted as traps for neutralizing antibodies, while full capsids could freely infect target cells. To avoid toxicity, the authors mutated the AAV2 capsid protein, preventing it from entering cells. Excess empty capsids successfully delivered AAV2, 5, 6, and 8 vectors to passively immunized mice [69]. Despite promising data obtained in experimental wild-type mice and rhesus macaques, translating this approach to patients with severe diseases raises safety concerns. Adding empty capsid traps to full capsids will increase the overall AAV payload and, most importantly, liver transduction. Fatalities associated with liver failure have recently been reported with several different high-dose AAV applications [9]. Given the enormous doses used in some conditions (Table 1), further increases in viral load could significantly increase the incidence of adverse effects. Furthermore, empty capsids will elicit immune-complex-mediated responses similar to full capsids, thereby increasing the risks associated with such responses. Excess empty capsids may also compete with full particles for AAV receptors on target tissue and thus interfere with treatment efficiency.

The use of natural AAV serotypes as gene therapy vectors is associated with the risk of their recognition by antibodies and cells formed after natural immunization. Therefore, modified capsids should be more promising in this regard. For example, AAV serotypes obtained from primates are considered to be less known to the human population [70]. Synthetic or rationally designed capsids and chemical capsid modifications are also developed to evade recognition by existing immune system effectors [71]. In 2017, modified viral vectors based on AAVrh10 were obtained using rational design. These modified vectors had a higher rate of penetration into the nucleus and a higher level of transduction in vitro and in vivo compared to wild-type viruses. The resulting vectors were able to infect liver cells in the presence of neutralizing antibodies to AAVrh10 [72]. The use of the mutant AAV2(Y-F) capsid, which is virtually not destroyed by proteasomes, preserved transgene expression and largely avoided hepatotoxicity [73].

New approaches to the development of engineered capsids are based on the modification of the most immunogenic epitopes of capsids. Data from healthy population analysis and that collected during clinical rAAV applications may help to define immunodominant regions in widely used AAV capsids. Mietzsch and colleagues performed structural analysis of the interactions of human-derived neutralizing antibodies from three patients treated with the AAV9 vector Zolgensma, utilizing high-resolution cryo-electron microscopy [74]. The cryo-electron microscopy maps revealed the capsid–antibody contacts and the authors proposed amino acid substitutions to prevent antibody binding. Subsequently, these variants were tested for their ability to escape the mAbs. Introduction of six substitutions (hAEV6-Q588R) reduced the endpoint titer determined for human sera of six Zolgensma patients ~13-fold when compared to the AAV9 capsid [74]. Generating new capsids is a potential way to selectively target desired cell types and overcome existing immunity. The development of new capsid variants using rational design, directed evolution, and artificial intelligence is becoming increasingly common. Given the described immunogenicity risks, priority should be given to developments that include B- and T-cell epitope prediction [75] and deimmunization algorithms [76]. Also, several aspects should be monitored during development. Potential limitations of vector engineering relate to transduction efficiency, safety, and manufacturing issues. First, amino acid substitutions in capsid proteins can affect recognition not only by circulating antibodies but also by AAV receptors and co-receptors, potentially altering biodistribution [77]. Furthermore, the changes can result in the formation of peptide sequences that are themselves potent immunogens, which can further provoke the immune system and act as adjuvants. Another limitation is that changes to capsid proteins can affect the efficiency of AAV production, making the already expensive AAV production process prohibitive.

Masking of vectors from circulating antibodies with immunologically inert proteins may also be useful. For example, pre-incubation with human serum albumin (HSA) was reported to increase the transduction capacity of the liver in a hemophilia B model [78]. In this work, the natural ability of AAV to bind HSA was exploited. Later, an engineered albumin-binding consensus domain (ABDCon) peptide was incorporated into the AAV9 capsid via fusion to the N-terminus of the AAV9 VP2 capsid protein to generate a variant AAV9 capsid with albumin-binding properties [79]. This modification increased liver transduction in mice after intravenous injection. Similar modification of VP3 protein was proposed in a recent paper [80]. In contrast to VP2 modification, VP3 protein modification will result in incorporation of 60 instead of 5 albumin-binding peptides. The author put forward a hypothesis that such modification will provide masking to enable administration of AAV vectors in the presence of high-titer antibodies including serial re-dosing of the same vector. Based on known affinities of albumin-binding peptides with HSA, HSA concentration in blood, immunoglobulin concentrations in circulation, and AAV2 primary receptor affinity, authors have provided rationale of feasibility of the approach. At the same time, it is necessary to solve in practice such technical issues arising after capsid protein modification as the stability of modified particles, in vivo half-life, the productivity of vectors, the packaging of the vector genome, transduction into target cells and biodistribution, as well as their protective capabilities against pre-existing antibodies [71]. The aforementioned studies experimentally investigated only the potential for transduction through the liver, while further testing is required for other targets, such as those in neuromuscular diseases. HSA masking, which protects against Ig recognition, may also hinder AAV binding to the target receptor, thereby significantly reducing transgene delivery.

## 4. Necessity of Repeated Injections

At present, the need for repeated administration has not been proven for hereditary diseases, since the observation period for patients after injection does not exceed 10–15 years. Most likely, repeated administration will be required for patients with certain diseases. Several factors such as target tissue, type of disease, and affected protein characteristics should determine this need.

### 4.1. Characteristics of Target Cells

Vectors based on adeno-associated viruses provide delivery of a single-stranded genetic construct, which includes the gene of interest and regulatory regions, to the nucleus of the target cell. In the nucleus, the second chain is synthesized and circular concatemeres are formed or, in rare cases, integrated in a known locus on the chromosome. Throughout the life of the cell, the required gene therapy molecule will be synthesized from the persistent episome. During cell division, the ring episomes are randomly distributed between daughter cells, resulting in a decrease in the load of gene therapy genes in the nucleus. Over time, the target “treated” cells will not be able to provide a sufficient level of expression of the therapeutic protein. Episome dilution has indeed been observed in pre-clinical studies of gene therapy drugs. For example, a knockout mice model of Crigler–Najjar syndrome showed a 10-fold reduction in viral genomes in the liver from 15 to 150 days of age after a single injection into mice at P2 [81] or reported luciferase gene expression decreased to 61.9% on day 28 [82]. Recent studies on long-term durability of transgene expression in hemophilia A dogs demonstrated up to 10 years of transgene expression after AAV8- and AAV9-FVIII delivery [83,84]. Moreover, the use of liver-targeted viral vectors in adult patients demonstrates stable expression levels over several years [85,86]. Thus, only clinical trials in the pediatric population can provide the necessary data for or against the transgene dilution in growing organs.

For highly differentiated cells such as neurons, a single injection of therapeutic AAV should provide lifelong expression. Highlighting the remarkable durability of Zolgensma, data from LT-001, an ongoing 15-year study, showed that up to 7.5 years post-intravenous dosing, children treated after presenting symptoms of SMA maintained all previously achieved motor milestones [87,88].

Muscle tissue as a target organ for gene therapy has its own characteristics due to the structure of the mature myotube. Because of multinuclearity, the presence of therapeutic episomes in only some nuclei can compensate for the expression of the therapeutic protein. Moreover, muscle growth and regeneration can also provide sufficient protein expression if at least some transduced myoblasts are involved in the fusion. On the other hand, terminally differentiated myotubes undergo necrosis during the pathogenesis of, for example, Duchenne muscular dystrophy, which can cause gradual transgene loss. Muscle tissue makes up to 40% of body weight and the drug injection occurs before the period of intensive growth and muscle mass gain. How the expression will be maintained after adolescence is still unknown. Currently, the stability of microdystrophin expression in skeletal muscle biopsies has been confirmed for 1 year after systemic injection [89], and the functional effect is maintained over 5 years of follow-up [90].

Monitoring patients receiving the first AAV-based therapies approved for clinical use provides invaluable data on the duration of transgene expression in various tissues and across various tissues, vector serotypes, and doses [91]. A thorough analysis of these results will aid in understanding existing correlations and using them to improve future rAAV designs and clinical trial protocols.

### 4.2. Characteristics of Disease and Therapeutic Protein

The need for repeated administration of the rAAV drug may be dictated by the characteristics of the disease and the therapeutic protein. In the case of enzyme or protein factor deficiency, a certain level of expression must be maintained for the therapeutic effect. For example, acid α-1,4 glucosidase (GAA), the deficiency of which causes Pompe disease, a lysosomal storage disease, has a short half-life of approximately 2–4 days [92]. At the same time, expression levels several times higher than normal are required to ensure a systemic effect [93]. Thus, a gradual decrease in the expression level of a therapeutic protein may be more detrimental in diseases caused by enzyme or short-lived protein deficiencies. It was reported that GAA blood levels decreased over time by 1.5–2-fold from the peak in two of three patients with Pompe disease who received AAV8-GAA in the phase 1 clinical trial conducted by Asklepios Biopharmaceutical [94]. Contrarily, structural proteins like dystrophin can persist for longer, with a half-life exceeding 100 days [95]. Multiple observations from animal studies and patient data demonstrate that even a low level of dystrophin can be beneficial for functional recovery [96].

Some diseases are caused by heterozygous mutations, in which one of the alleles contains a mutation that disrupts protein synthesis. The pathogenesis of the disease in such cases is explained by haploinsufficiency and is typical for dose-dependent genes. For example, hypertrophic cardiomyopathy can be caused by heterozygous mutations in the *MYBPC3* gene. In pre-clinical studies, it was found that the effectiveness of therapy directly depends on the level of protein expression in individual animals [97]. For such diseases, a decrease in the level of expression of the target protein can have a greater impact on therapy efficacy than for others.

The need for repeated administration of rAAV products may also stem from the necessity to administer doses sufficient to achieve efficacy in clinical trials. To ensure safety, trials typically use escalating doses of the products. In this case, patients who have previously received ineffective doses are no longer eligible for treatment because they have developed lifelong immunity to both the vector and the transgene.

## 5. Ways to Overcome Immune Response for Repeated Delivery

Given the complex nature of the immune response to rAAV-based drugs described previously, as well as the reasons for requiring a second or subsequent injection, and the characteristics of pre-existing immunity, ensuring safety and maintaining efficacy with repeated injections is challenging. In simple terms, barriers to re-administration of AAV are circulating binding and neutralizing antibodies, producing plasma cells as well as memory B cells and, on the other hand, memory T cells, the activation of which triggers a cytotoxic cell response. Here we divide strategies aimed at minimizing the immune response into two categories: manipulations of the first injection (Section 5.1) and manipulations of the second injection only (Section 5.2). These two approaches, while very similar in complexity, differ in their applicability to different patient groups and the methods of their implementation in clinical practice. Manipulations of the first injection allow for profound modification not only of the rAAV drug itself but also prepare the patient for the first antigen exposure. In contrast, manipulations of the second injection are limited by input factors and are further complicated by individual patient characteristics and variations in established immune memory.

### 5.1. Minimization of Immune Response During First Injection

The vast majority of studies in laboratory animals investigating repeated administration of rAAV-based drugs have examined delivery methods in which manipulations with the drug and/or the animal are performed prior to the initial exposure. This approach allows researchers to mask the drug’s components from the innate immune system and inhibit the formation of immunological memory. Currently tested strategies can be grouped as follows: the first group includes modifications of the drug itself (Section 5.1.1), and the second group includes the use of pharmacological agents administered before, during, and after administration of the drug (Section 5.1.2). Both areas should be considered as a priority and taken into account when developing new AAV-based gene therapies to improve their safety profile and enable the potential for re-injection if needed in the future.

#### 5.1.1. Optimization of Genetic Construct

Special attention is given to the design of the therapeutic construct. Reducing immunogenicity through vector design relies on sequence optimization to avoid recognition by the innate immune system and selection of expression control elements that minimize the presence of the therapeutic protein in non-target tissues, particularly antigen-presenting cells.

Unmethylated CpG motifs are common in AAV vectors due to hypomethylation of vector genomes during their production and the presence of cross-packaged microbial-derived genetic constructs that are rich in CpG. Reducing the number of CpG motifs in AAV vector genomes is essential to enhance the safety profile of the final product [15,98]. An approach to quantify the TLR9 activation potential of different sequences has been proposed [99]. Modification of the majority of trigger sequences through codon optimization can be carried out using automated computer algorithms [99]. At the same time, introducing rare codons should be avoided, as this can significantly reduce protein expression levels. Conversely, using preferred codons can increase expression levels at the same vector dose, as demonstrated in pre-clinical trials [45,81]. The use of tissue-specific promoters is widely employed in gene therapy constructs (Table 1). Additionally, tissue-specific enhancers, inducible promoter elements, and miRNA-binding sites can be incorporated to reduce transgene expression in non-target tissues [100,101,102]. Enhancement of transgene expression with additional cis-acting elements, such as introns and post-transcriptional regulatory elements, allows for a lower overall dose and thus an improved safety profile. The advantages of such elements have been discussed in a recent review by Wang and colleagues [102].

#### 5.1.2. Pharmacological Assistance

**Figure 3:**
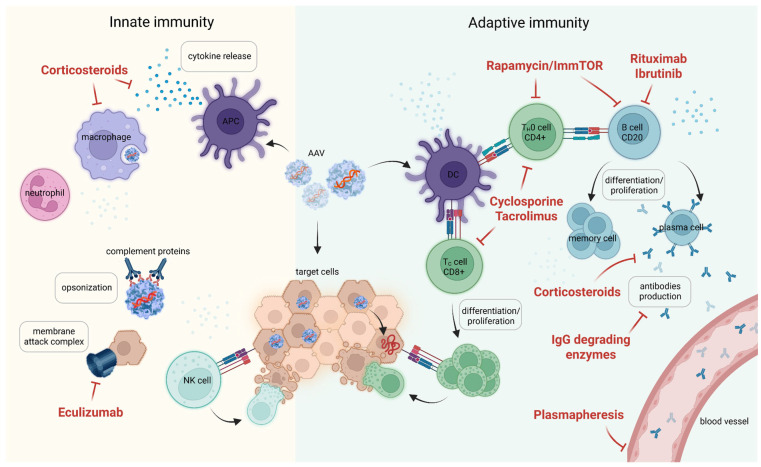
Pharmacological approaches to minimize immune activation during first AAV injection. Pre-clinical strategies to mitigate anti-AAV antibody responses include the use of immunosuppressants (rituximab, cyclosporine A), immune-tolerizing therapies (tolerogenic rapamycin nanoparticles, ImmTOR), IgG-specific proteases, and IgG depletion through plasmapheresis or affinity absorption. Created in Biorender. Egorova, T. (2025) https://BioRender.com/ql2bipu (accessed on 17 July 2025).

Immune responses to the vector and protein that develop during high-dose viral administration were the cause of transgene loss in early clinical studies. This has spawned an entire field of research to resolve the acute immune response and to select pharmacological agents that allow successful expression of the therapeutic protein (Figure 3). To date, patient inclusion criteria and protocols for managing immune reactions to AAV infusion have been established, enabling the achievement of stable expression of therapeutic constructs in various organs and tissues. These immunosuppression regimens are aimed at blocking immediate reactions but do not prevent the formation of humoral and cellular immunity during the first injection. Extended immunosuppression schemes could potentially limit immunologic memory development and facilitate a second infusion if necessary. In recent years, animal studies testing two consecutive doses of AAV have been actively conducted.

Rapamycin is a general immunosuppressant that inhibits T- and B-cell activation and leads to a decrease in the production of antibodies to AAV. Its mechanism of action involves inhibiting the mTOR pathway and subsequently modulating the immune response. Widely used in organ transplantation to prevent rejection, rapamycin suppresses B-cell proliferation and differentiation; at high doses, it also inhibits cytotoxic T-lymphocyte (CTL) and T-helper cell activation [103,104]. Different groups tested the potential of rapamycin in combination with other immunosuppressants for inhibiting immune response during AAV delivery [105,106,107,108]. The most valuable effect on immune suppression was achieved when using rapamycin encapsidated into biodegradable nanoparticles: PLGA [109], SVP (PLGA, PLA-PEG) [110,111]. Ilinsky and colleagues used secreted embryonic alkaline phosphatase (SEAP) as a reporter, which was delivered by AAV8 in the presence of SVP–rapamycin nanoparticles designated ImmTOR. ImmTOR accumulates in the spleen and liver where it is endocytosed by antigen-presenting cells along with the antigen. Antigen presentation in the presence of rapamycin induces a tolerogenic environment that blunts the adaptive immune response. The authors repeatedly treated mice with AAV-SEAP mixed with ImmTOR nanoparticles and demonstrated serum SEAP level upregulation in the presence of ImmTOR after first and second AAV dosages. ImmTOR admixing protected AAV particles from neutralization with nAbs with low neutralizing activity during passive immunization. These results suggest the feasibility of AAV-mediated transgene delivery in seropositive patients when appropriate immunosuppression is used. To check the influence of adaptive immunity on enhanced transgene expression the authors used Rag2-KO mice lacking T and B lymphocytes. Repeated administration of AAV8-SEAP into Rag2-KO mice resulted in enhanced SEAP expression in contrast to wild-type mice. ImmTOR addition gave even higher SEAP expression rates. Thus, the beneficial effect of ImmTOR on first dose transgene expression appears to be independent of its immunomodulatory effects on adaptive immunity. The main drawback of the proposed discovery is the low AAV dose used for the study. Successful re-administration was demonstrated for the 5 × 10^11^–2.5 × 10^12^ vg/kg dose range. Using the SEAP reporter gene does not allow for assessing the expression level of the therapeutic transgene. The level achieved with this dosing regimen may be insufficient to modify the disease course. Currently approved drugs necessitate doses of 5 × 10^11^–1.33 × 10^14^ vg/kg for systemic injections [112]. In a recent paper authors explored the potential of the combination of ImmTOR nanoparticles with B-cell-targeting drugs for the ability to increase the efficiency of repeated administration at high vector doses. These included a monoclonal antibody against B-cell activation factor (aBAFF) and Bruton’s tyrosine kinase inhibitor, ibrutinib. The authors demonstrated strong synergy of drugs leading to a more than 5-fold to 10-fold reduction of splenic mature B cells and plasmablasts [113].

In their work, Choi and colleagues paid particular attention to humoral immunity. They used a combination of the proteasome inhibitor bortezomib and anti-CD20 antibodies for B-cell depletion from blood, spleen, and bone marrow and prevented the formation of anti-AAV8 nAbs. Sixteen-week-long immunosuppression guaranteed the highest hSEAP expression following two subsequent AAV8 injections [114]. Similarly, implementation of a combination of monoclonal Ab therapies against CD20 and BAFF allowed successful AAV8 gene transfer to the liver and hFIX transgene detection in circulation [115].

The study presented by Liao and colleagues focused on the possibility of repeated high-dose administration [82]. The authors proposed to modify rapamycin and dexamethasone by chemically linking them to lipid carriers, allowing co-assembly into stable nanoparticles measuring 100–180 nm under physiological conditions. This nanoparticle formulation combines the inhibitory effect of rapamycin nanoparticles on neutralizing IgG formation with dexamethasone’s impact on IgM expression and complement activation. In vitro studies have demonstrated great potential of these nanoparticles to inhibit complement activation and reduce the expression of proinflammatory cytokines IL-6 and TNF-alpha. During two subsequent injections of AAV8 bearing luciferase and SEAP reporter genes, the authors observed complete inhibition of IgG and IgM secretion, along with a reduction in germinal center and T-follicular helper cell numbers and the induction of immunosuppressive Treg cell expansion. More importantly, co-administration of the nanoparticles enabled increased transgene expression after a single injection and following repeated high-dose (1.2 × 10^13^ vg/kg) delivery of AAV8-SEAP. However, in the this study, the authors did not explore the potential of the engineered nanoparticles for gene delivery in the presence of pre-existing immunity, which would be a great contribution for future studies [82].

In 2012 Mingozzi and colleagues reported successful coagulation factor IX expression following AAV6 injection into rhesus macaques pre-immunized with AAV8. This was possible after immunosuppression with the combination of the anti-B-cell monoclonal antibody rituximab (Rtx) in combination with cyclosporine (CsA) [116]. Rituximab-induced B-cell depletion in combination with cyclosporine, a selective T-cell suppressor, results in a reduction of neutralizing antibodies produced in response to the first AAV injection. However, this immunosuppressive protocol, although safe, is less effective in the case of high nAb titers. The reason that Rtx does not always reduce circulating anti-AAV antibody levels may be that this anti-CD20-depleting antibody does not target CD20-negative plasma B cells, so administration of the drug does not result in complete depletion of B cells in lymphoid organs.

Non-human primates are the most relevant animals for immune response studies. Unzu and colleagues tested a robust immunosuppression regimen during rAAV5-mediated liver-targeted porphobilinogen deaminase gene delivery. The authors used a combination of the B-cell-depleting rituximab, antithymocyte gamma globulin, methylprednisolone, mycophenolate mofetil, and tacrolimus. Antithymocyte gamma globulin (ATG) is a polyclonal antibody against T cells and their precursors, thymocytes. This drug is used as an immunosuppressant in the treatment of aplastic anemia, as well as in organ and stem cell transplants [117]. Methylprednisolone, a corticosteroid, is used in immunosuppressive protocols to inhibit immune responses to AAV by reducing proinflammatory cytokines/chemokines and attenuating liver toxicity. Mycophenolate mofetil inhibits T- and B-cell proliferation by targeting type II inosine monophosphate dehydrogenase, thus suppressing both cell-mediated and humoral immune responses. Tacrolimus, like cyclosporine, is a calcineurin inhibitor that results in calcium-dependent inhibition of T-cell activation [118]. Authors demonstrated a delay and attenuation of rAAV5 capsid immunity in immunosuppressant-treated animals. However, the five-drug combination failed to allow the delivery of eGFP reporters using the same vector. Moreover, the authors found inhibition of liver transgene expression while the animals were immunosuppressed with mycophenolate mofetil. The mechanism of such interference remains to be elucidated [119]. Regeneron reported successful AAV re-administration in non-human primates (NHPs) using two different immunosuppression regimens at the ASGCT2024 annual meeting. The presentation covered a comparison between a novel strategy using bispecific antibodies and a more traditional strategy using rituximab. Bispecific antibodies have been proposed as a temporary therapy designed to maintain seronegativity only during key periods of treatment, potentially allowing for flexible and repeatable gene therapy [29].

Immunosuppression protocols came to the gene therapy area from oncology and transplantation. In current clinical practice, agents such as different corticosteroids, tacrolimus, mycophenolate mofetil, cyclosporine, sirolimus, and rituximab are used to manage immune-mediated adverse events that may be developed during or after rAAV gene therapy [118]. The side effects of proposed regimens are well established and lead to increased susceptibility to infectious diseases. In gene therapy applications, side effects of immunosuppression may depend not only on the drug action but also on the course of the genetic disease itself and its stage of development. A treatment regimen that is safe for one disorder may be intolerable for another. Careful monitoring and appropriate prophylactic measures are essential to minimize the risk of infections in patients receiving immunosuppressive treatments.

The approaches described in Section 5.1 can prevent the immunogenicity of AAV-based drugs, however, these strategies have both advantages and limitations. The feasibility of using a particular method to reduce the immune response to AAV is influenced by the presence of pre-existing immunity [68]. A comparison of different strategies is presented in Table 2.

### 5.2. Manipulations Only with Second Injection

Research into suppressing the formation of immune memory following initial exposure to AAV is of great interest and will pave the way for future gene therapies. At the same time, a significant group of patients have already received their first doses of AAV in clinical trials or in real-world clinical practice. For example, over 4000 patients worldwide have been treated with Zolgensma [128] and hundreds of patients with hemophilia have received rAAV [129]. In cases of transgene loss and gradual waning of therapeutic efficacy, these patients have alternative treatment options. For the majority of patients who have received ineffective doses of rAAV drugs or who did not respond to rAAV treatment in clinical trials, no alternative treatment options exist. For these patients, newly developed rAAV drugs may represent the only chance for a cure and pre-existing immunity acquired during initial exposure to AAV can prevent them from receiving life-saving therapy. Therefore, exploring strategies for re-administration in patients with pre-existing natural immunity to the vector or who have already received therapeutic rAAV is crucial. Most current studies focus on depletion of pre-existing antibodies to AAV vectors in circulation. These approaches include plasmapheresis [121,122], immunoadsorption [123], AAV-binding Ig depletion [120], and the use of IgG-degrading enzymes [124,125].

In seropositive NHP, two consequent cycles of apheresis resulted in 1.5–120-fold reduction of AAVrh74-binding antibody levels. This allowed expression of uDys similar to those obtained in seronegative NHPs treated with AAVrh74-uDys [121]. In another study, the feasibility of using an immunoadsorption (IA) procedure for repeated, liver-targeted gene delivery in non-human primates was explored. The animals were administered IV with rAAV5 carrying the hSEAP reporter gene. Seven weeks after the first rAAV treatment, all of the animals were re-administered with rAAV5 carrying the therapeutic hemophilia B gene human factor IX (hFIX) and underwent IA prior to the second rAAV5 exposure. Although no hFIX was detected after rAAV5-hFIX re-administration without prior IA, all animals submitted to IA showed therapeutic levels of hFIX expression. Additionally, a threshold of anti-AAV5 nAb levels compatible with successful re-administration was demonstrated. The analysis of anti-AAV nAb levels in human subjects submitted to IA confirmed the safety and efficacy of the procedure to reduce anti-AAV nAbs [123]. This study demonstrates encouraging results from repeated administration of gene therapy in primates and the possibility of achieving therapeutic levels of the transgene after a second injection. Since the use of IA results in a strong decrease in the total IgG level, a sufficient threshold titer of neutralizing antibodies for each serotype should be determined to ensure a safe balance between second administrations and the preservation of the patient’s immune system.

Plasmapheresis and protein-G-based immunoadsorption result in non-specific depletion of immune molecules and may lead to more severe immunodeficiencies; therefore, specific methods are preferred. A method of AAV-specific circulating antibody depletion was proposed by Orlowski and colleagues [120]. The authors immobilized AAV9 particles on sepharose beads and used them for immunoadsorption. They clearly demonstrated depletion of AAV-specific antibodies during hemapheresis. Additionally, they have shown that, after nAbs depletion, the total IgG levels remain unchanged, which distinguishes this method from protein-G-based plasmapheresis. Anti-AAV9 antibody depletion in passively immunized rats allowed for successfully delivering the luciferase transgene into the liver and heart [120]. Targeted depletion of AAV-specific antibodies holds great promise due to its specificity and limited impact on protection against other pathogens. Potential limitations relate to the production of ready-to-use sorbents for clinical use, their stability, and standardization.

There are a few works utilizing immunoglobulin-G-degrading enzyme of Streptococcus pyogenes (IdeS) infusions in laboratory animals [124,125,126]. Leborgne and colleagues demonstrated that IdeS pre-treatment (500 mkg/kg), administered two days before AAV delivery into seropositive NHPs, results in higher transduction efficiency (VGCN/diploid genome) and increased transgene expression. However, when the authors performed repeated AAV-hFVIII injections into an IdeS-pre-treated NHP group, they demonstrated only transient elevation of FVIII in circulation. The researchers explain this phenomenon by appearance and action of anti-hFVIII antibodies. It is promising work about IdeS pre-treatment of seropositive recipients that prevents AAV neutralization and allows successful transduction of target tissues. Further studies exploring the combination of IdeS with immunosuppressive drugs are highly awaited to prevent transgene clearance caused by neutralizing antibodies and T-cell immunity to the transgene [124].

Recent work in this area suggests using an engineered IgM-cleaving enzyme (IceMG) with dual endopeptidase activity specific to both IgM and IgG antibodies. Upon intravenous dosing, IceMG rapidly and reversibly clears circulating IgM and IgG in rhesus macaques. Antisera from these animals treated with IceMG show decreased AAV neutralization and complement system activation. Consistently, pre-conditioning with IceMG of mice passively immunized with human antisera restores AAV transduction at a dose of 1 × 10^13^ vg/kg dose [127]. The advantage of IgG-degrading enzymes is that they may involve less invasive procedures compared to hemapheresis.

## 6. Future Perspectives

Pre-existing immunity to AAVs significantly limits gene therapy efficacy and patient eligibility. Advances in capsid engineering, immune modulation, and alternative delivery methods are essential for expanding access to treatment. Clinical trials are actively exploring these approaches to ensure safer, more effective gene therapies. The possibility of repeated administration of adeno-associated-virus-based drugs has been a concern for scientists since the very beginning of their development. However, an effective solution has not yet been found, which is due to the lack of fundamental research and knowledge in the field of formation of immunity to AAV. Various approaches to repeated delivery of transgenes using AAV have been tested in animal models of various diseases, including titer increase [130,131], serotype change [132,133], the use of immunosuppression during the first and subsequent administrations [134], use of enzymes that break down antibodies, and plasmapheresis [135]. However, using animal disease models to assess immune responses has limitations, as the pathogenesis is not always reproduced in animals, which can significantly influence immune status or viral biodistribution [136]. In this regard, new models must be created and adequate protocols developed that will bring physiology closer to the patient. Mice, the most common experimental animal models, are not a natural host of AAV and cannot fully reproduce antibody-mediated immune responses. As pointed out in a number of clinical trials, complement-system-triggered immune reactions failed to arise in these animal models. To overcome the differences in immune system activity and find a way to manage complement system activation consequences, repeated AAV administration in mice was proposed [131]. Administration of a high dose of AAV in the presence of specific antibodies to AAV formed after the first injection in mice induced complement activation and expression of proinflammatory cytokines of myeloid origin, as observed in some clinical studies. Thus, optimized testing conditions will help to predict potential adverse reactions and prevent them in future clinical protocols. Despite promising results in pre-clinical studies, no approach to evading the immune response during repeated administration of AAV gene therapy has been applied in clinical trials. This again highlights the existing challenges of moving forward from pre-clinical models to clinical testing on patients. The only registered clinical trial aims to investigate the potential of repeated administration of AAV in Pompe disease (NCT02240407) [137]. The results of this study have not yet been reported.

Since B-cell-mediated immunity is a critical component in preventing vector re-challenge, one possible avenue to reduce the immune response against AAV is to use simultaneous depletion of IgG and IgM antibodies. It was found that IgG depletion alone is not sufficient to allow vector re-challenge, suggesting an important role for IgM antibodies in the immune response against AAV. In addition, IgM antibodies are more prone to complement activation than IgG antibodies. Mouse models lacking IgM heavy chains may prove useful for future gene delivery studies, as they can be used to study repeated dosing of AAV-based drugs [82,138]. Protocols for IgG depletion or degradation have demonstrated potential for facilitating AAV re-challenge in laboratory animals. However, strategies to block cellular immunity to AAV have so far been tested only for primary AAV exposure. In the examples provided in Section 5.1.2, tacrolimus and cyclosporine are used to inhibit T-cell proliferation and limit cellular responses. The combination of this pharmacological approach with IgG depletion should be further explored in pre-clinical settings to identify a safer method for AAV re-administration.

Pharmacological immunosuppressive treatments, including pre- and post-injection protocols, impose a significant burden on patients. The underlying genetic diseases are often associated with increased susceptibility to viral and bacterial infections due to weakened respiratory muscles in conditions, like Duchenne muscular dystrophy or spinal muscular atrophy, or altered mucus viscosity in cystic fibrosis, among other factors. Additional immunosuppression can lead to severe infectious complications. Consequently, prolonged patient isolation is often recommended to minimize the risk of pathogen exposure. In contrast to traditional immunosuppression drugs, which maintain total immune suppression, ImmTOR induces tolerance only to specific co-delivered antigens, allowing the immune system to accept these foreign substances without overreacting, resulting in long-term tolerogenic responses. At the same time, ImmTOR maintains the immune system’s ability to defend against other pathogens and unrelated antigens. Therefore, the nanoparticle-based strategy has an improved safety profile when compared to convenient immunosuppression protocols. Similarly, methods for depleting AAV-recognizing antibodies are safer than total IgG depletion.

In addition to strategies targeting the immune system, recent reports have described modifications of capsid proteins that can improve transduction efficiency and help evade immune responses. Molecular engineering techniques (destruction of known epitopes; tyrosine mutation to limit ubiquitination and proteasomal processing) can be used to create new weakly immunogenic AAV capsids [3]. AI-based approaches could further improve the design of new capsids with desired characteristics, including the ability to evade the immune response [139]. However, it should be taken into account that new challenges may arise in the production and use of new genetically engineered capsids, including potential loss of transducing activity, unforeseen causal effects upon administration, and deterioration of AAV assembly, which can lead to decreased yield [140] and increased production costs.

Current regulatory guidelines require consideration of pre-existing immunity to vectors and potential immune responses to both the vector and the transgene. The recommendations also address the possibility of using immunosuppressive therapy to ensure gene therapy efficacy in pre-immunized patients or testing re-administration of the gene therapy drugs, which requires clinical trials. It is also noted that it is impossible to provide specific recommendations on the optimal drug combination for all gene therapies, as the choice of regimen will likely depend on the disease being treated and any associated complications [141]. The importance of studying potential reactions to ensure the efficacy and safety of AAV-based gene therapy products is underscored by the fact that the FDA has initiated its own research to develop highly sensitive test systems for monitoring the cellular and humoral response to AAV [142].

Based on the analysis of existing literature, we conclude that there is no universal solution for preventing immune responses to AAV or for administering AAV-based therapies to seropositive patients. This obstacle is complex and must be considered within the context of disease pathogenesis, the vector and transgene used, characteristics of the target population, and other factors. Simultaneously, the development of new, highly effective vectors to reduce overall viral load, optimization of genetic construct designs, and the selection of appropriate immunosuppressive protocols and manipulations lay the essential groundwork for successful re-administration of AAV-based therapies.

## Figures and Tables

**Table 2:** The comparison of strategies to reduce AAV immunity.

Proposed Strategy	Advantageous Aspects	Limitations	References
For Naive Patients During First AAV Treatment			
Reduction of CpG content by codon optimization of coding sequence	Reduces TLR9 activation	Can affect expression rate	[15,98,99]
Optimization of expression control (tissue-specific promoters and enhancers, miRNA-binding sites, introns, post-transcriptional promoter elements)	Reduces overall dose and increases expression in target tissues while limiting off-target expression	Can affect AAV packaging and genome integrity	[100,101,102]
Pharmacological inhibition of immune memory formation (B-cell depletion, proteasome inhibition, T-cell suppression)	Inhibits immune memory formation and allows next rAAV injections if necessary Using drugs already available in clinical practice	Causes immunodeficiencies and may worsen the condition of patients with certain genetic diseases	[29,82,114,115,116,119]
Nanoparticle-assisted strategies	Inducing immune tolerance to a particular vector and transgene which are co-injected without affecting defense against pathogens	Absence of approved drugs	[112]
For patients with pre-existing immunity or for repeated rAAV injections			
Empty capsids decoys	Reduces capturing of full capsids by pre-existing circulating antibodies	May provoke Ig-mediated immune reactions May reduce target tissue’s transduction due to competition for the AAV receptor Lack of methods for efficient empty capsid purification	[69]
Capsid engineering	Reduces capturing of capsids by pre-existing circulating antibodies	Capsid modification may affect rAAV production efficiency and biodistribution	[71,72,73,74]
Capsid masking by HSA, chemical linking, exosome packaging	Reduces capturing of capsids by pre-existing circulating antibodies	May affect biodistribution Additional step of manufacturing may affect rAAV yield	[78,79,80]
AAV-specific circulating antibody depletion	Reduces Ig-mediated AAV-clearance and Ig-mediated immune reactions	Does not influence cellular immune memory	[120]
Plasmapheresis and protein-G-based immunoadsorption	Reduces Ig-mediated AAV-clearance and Ig-mediated immune reactions	Causes immunodeficiencies and may worsen the condition of patients with certain genetic diseases Does not influence cellular immune memory	[121,122,123]
IgG- and IgM-degrading enzyme	Reduces Ig-mediated AAV-clearance and Ig-mediated immune reactions	Does not influence cellular immune memory	[124,125,126,127]

## Data Availability

Not applicable.

## Abbreviations

The following abbreviations are used in this manuscript:

AAV	Adeno-associated virus
CTL	Cytotoxic T lymphocyte
DMD	Duchenne muscular dystrophy
IceMG	IgM-cleaving enzyme
IA	Immunoadsorption
ImmTOR	Tolerogenic rapamycin nanoparticles
IFNs	Interferons
MHC	Major histocompatibility complex
nAb	Neutralizing antibody
PAMP	Pathogen-associated molecular pattern
PRR	Pattern-recognizing receptor
SMA	Spinal muscular atrophy
TLR	Toll-like receptor
